# Supplementary material for: The RhoA-ROCK1/ROCK2 Pathway Exacerbates Inflammatory Signaling in Immortalized and Primary Microglia
Source: Cells. 2023 May 11;12(10):1367. doi: 10.3390/cells12101367 (PMC10216802; doi:10.3390/cells12101367)
Supplement: Supplementary file 1 [file cells-12-01367-s001.zip › cells-2330201-supplementary.pdf]

Figure S1

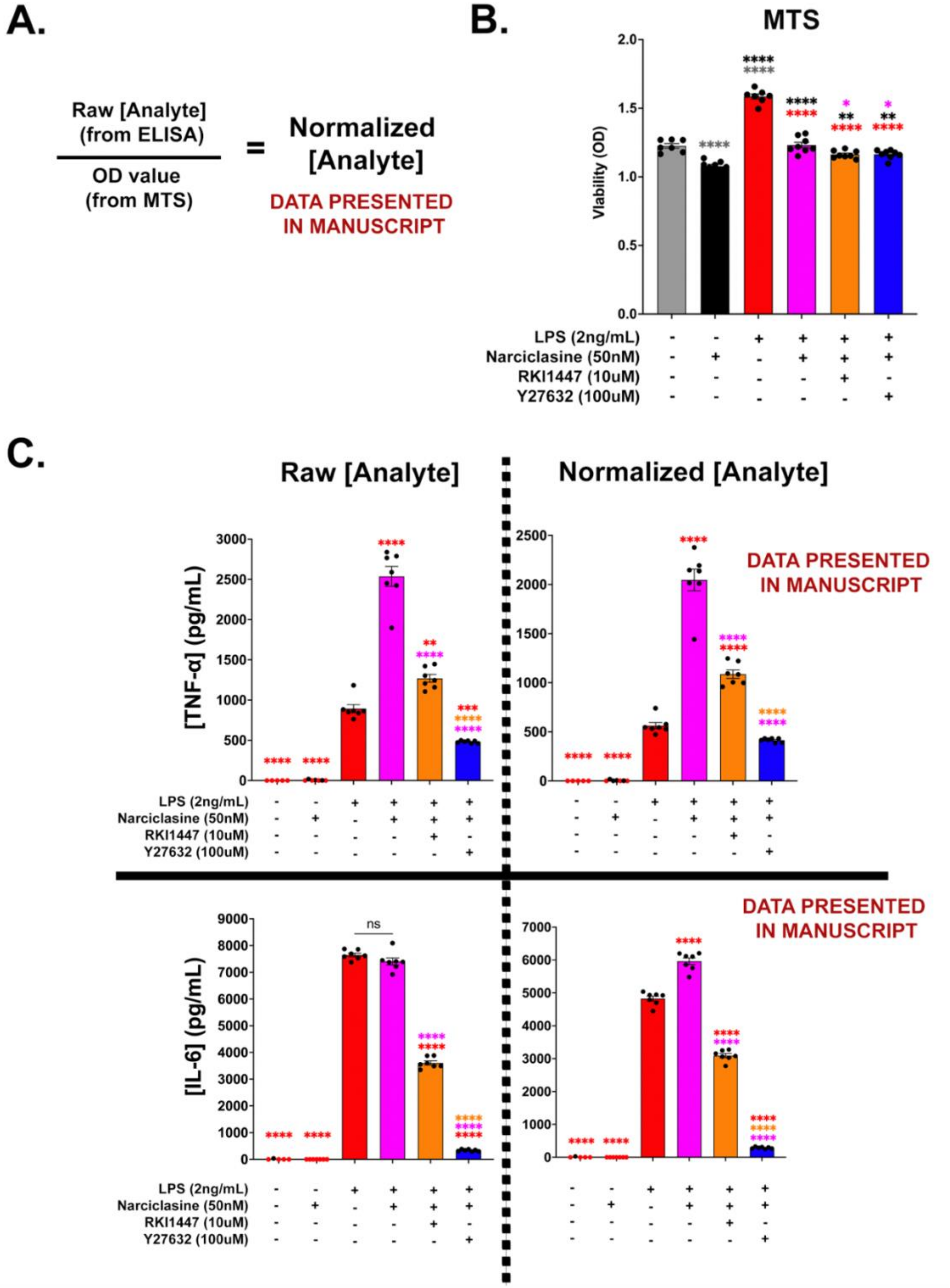

Figure S1. Example of normalization of raw ELISA data using the MTS vaibility OD value. (A) Calucluation used to determine normalized analyte concentration. These normalized analyte

concentration values are the data presented in the manuscript. (B) Absolute OD value from MTS assay shown in Figure 6E used to normalize data in Figure 6F. (C) Differences in raw ELISA data vs. normalized ELISA data from Figure 6F. When data is not normalized, no differences can be seen between [IL-6] in the LPS vs. LPS+Narc group.

Figure S2

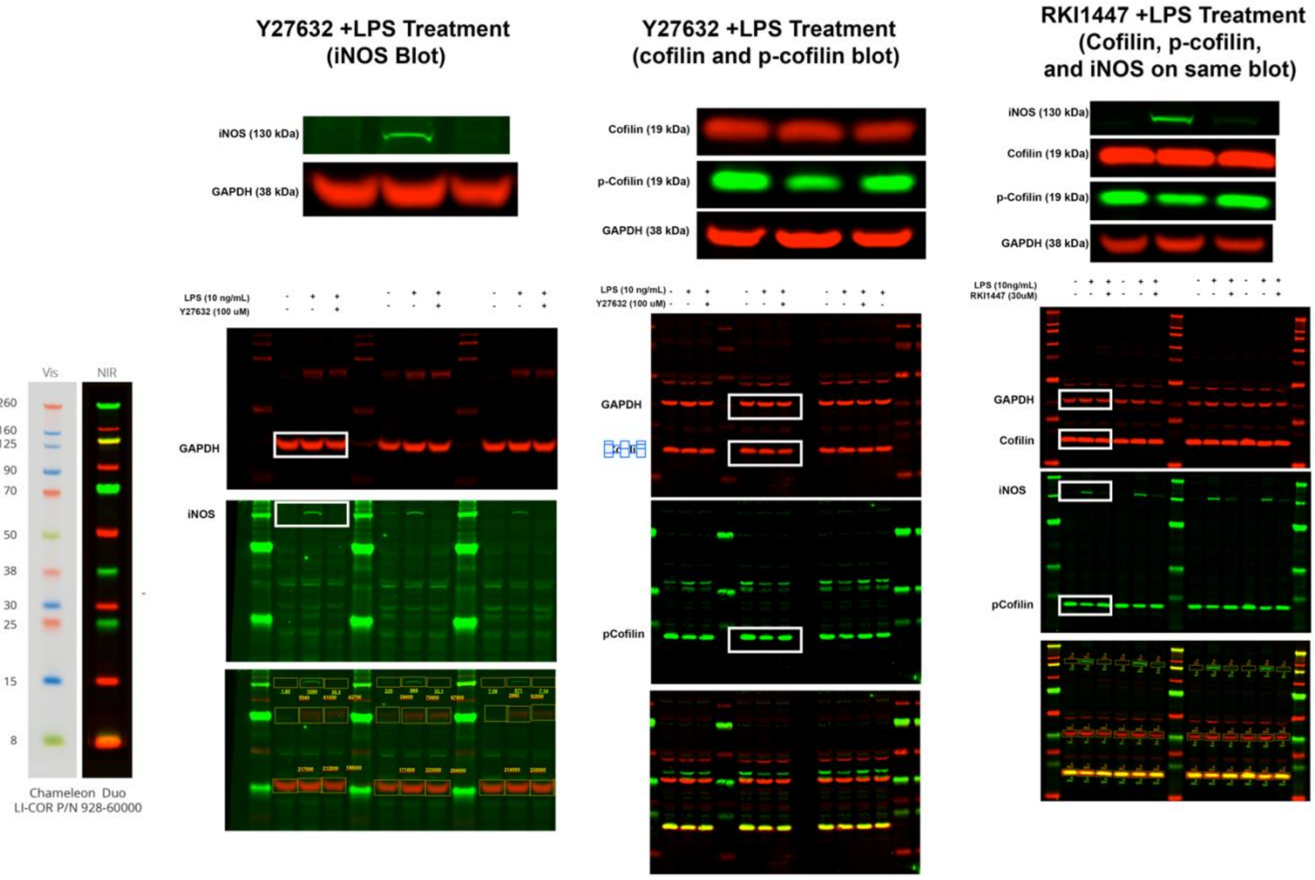

Figure S2. Full western blots with split and merged (680-Red and 800-Green) channels.

Figure S3

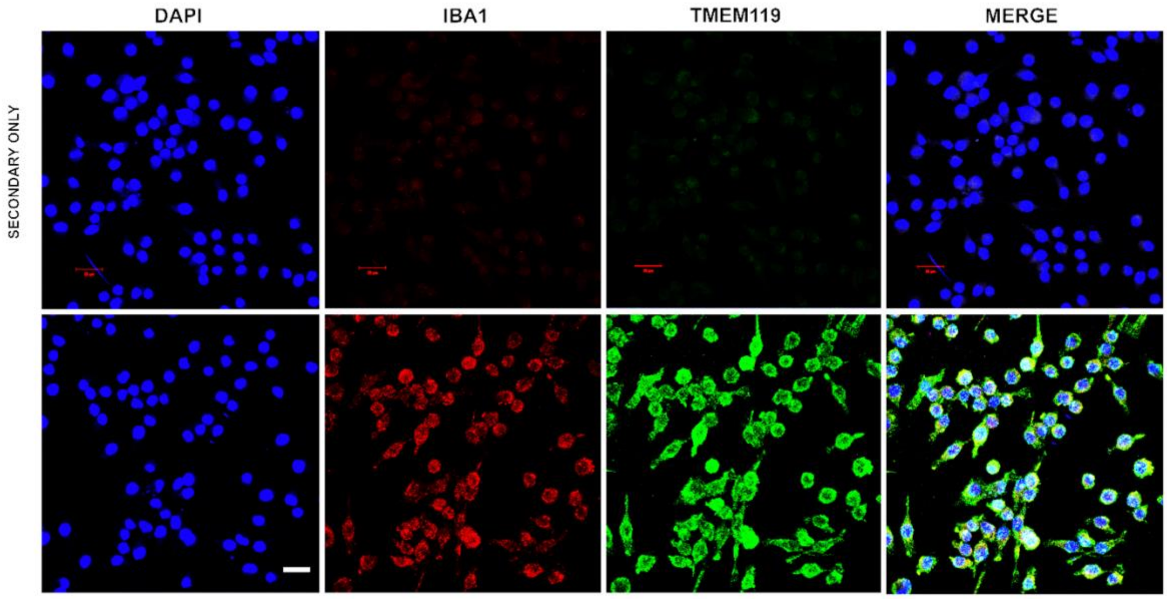

Figure S3. Secondary only controls for IMG cell staining.

Figure S4

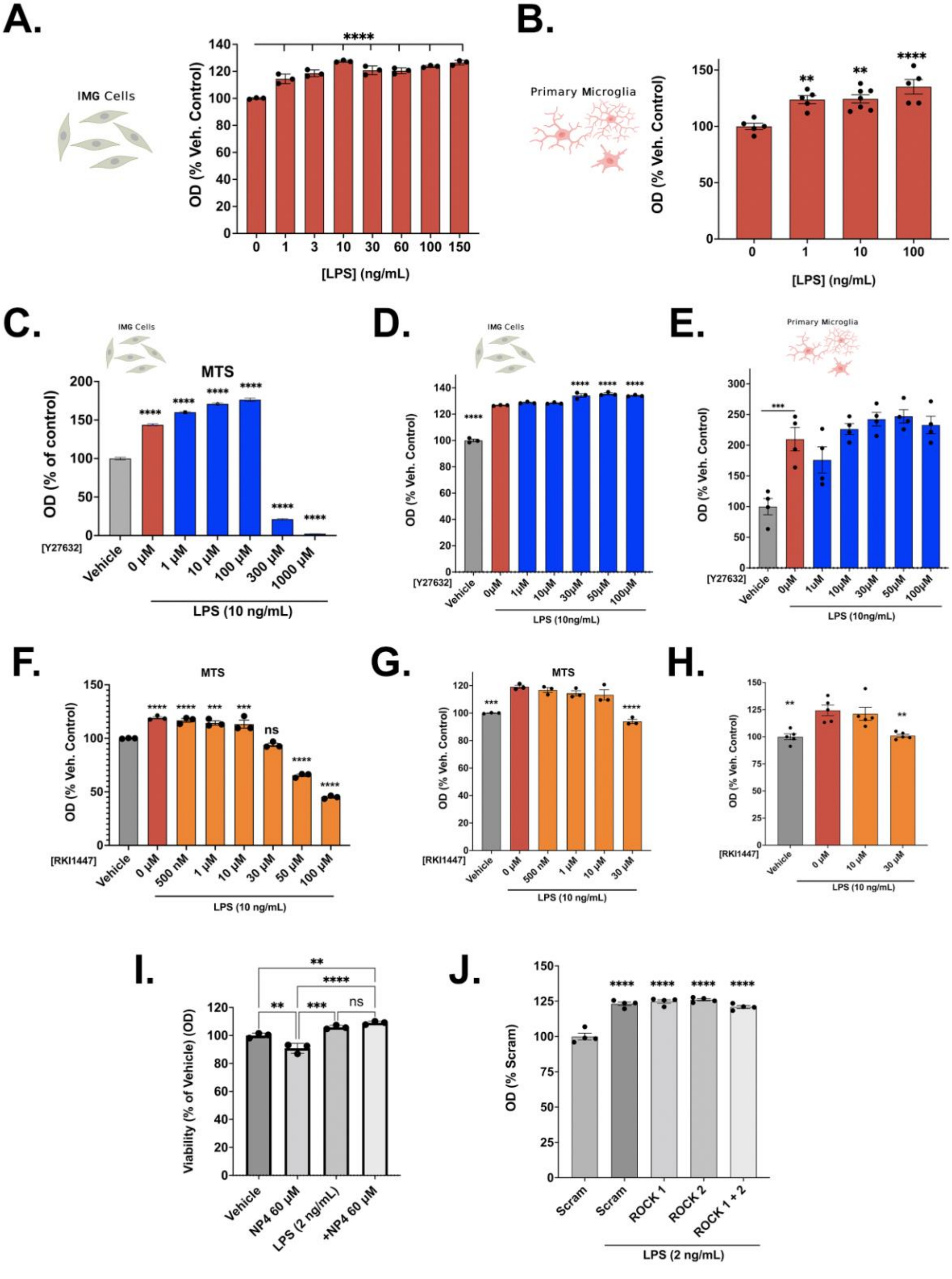
A.
IMG Cells
OD (% Veh. Control)
[LPS] (ng/mL)
B.
Primary Microglia
OD (% Veh. Control)
[LPS] (ng/mL)
C.
IMG Cells
MTS
OD (% of control)
[Y27632]
LPS (10 ng/mL)
D.
IMG Cells
OD (% Veh. Control)
[Y27632]
LPS (10ng/mL)
E.
Primary Microglia
OD (% Veh. Control)
[Y27632]
LPS (10ng/mL)
F.
MTS
OD (% Veh. Control)
[RKI1447]
LPS (10 ng/mL)
G.
MTS
OD (% Veh. Control)
[RKI1447]
LPS (10 ng/mL)
H.
OD (% Veh. Control)
[RKI1447]
LPS (10 ng/mL)
I.
Viability (% of Vehicle) (OD)
Vehicle
NP4 60 μM
LPS (2 ng/mL)
+NP4 60 μM
J.
OD (% Scram)
Scram
Scram
ROCK 1
ROCK 2
ROCK 1 + 2
LPS (2 ng/mL)

Figure S4. MTS assays used throughout studies for normalization of cytokine concentrations. (A) IMG cells and PMg (B) LPS dose response. (C) Toxicity of Y27632 with 10ng/mL LPS challenge establishing top dose (100 μM) used in subsequent studies. (D) IMG and (E) PMg Y27632 dose-response against LPS (10 ng/mL) challenge. (F) Toxicity of RKI1447 with 10ng/mL LPS challenge establishing top dose (30 μM) used in subsequent studies. (G) IMG and (H) PMg RKI1447 dose-response against LPS (10 ng/mL) challenge. (I) LPS (2 ng/mL) and Nogo-P4 (60 μM). (J) siRNA treatments vs LPS (2 ng/mL) challenge (cytokine assessment experiment). One-way ANOVA with Dunnett's (A-H AND J, compared to vehicle or LPS alone) or Tukey's (I) multiple comparisons was used for statistical analysis with error bars representing mean ±SEM; **=p<.01, ***=p<.001, and ****=p<.0001.
